# Cognition at the core of metabolic syndrome: linking metabolic load to behavioural impairment in a longitudinal high-fat diet rat model

**DOI:** 10.1016/j.bbih.2026.101287

**Published:** 2026-06-16

**Authors:** Nicolò Ricciardi, Valentina Di Liberto, Danila Di Majo, Antonio Cangelosi, Miriana Scordino, Giulia Urone, Giuseppe Giglia, Alessandro Massaro, Mario Allegra, Maria Ankarcrona, Pierangelo Sardo, Giuseppe Ferraro, Giuditta Gambino

**Affiliations:** a Department of Biomedicine, Neuroscience and Advanced Diagnostics (Bi.N.D), Section of Human Physiology, University of Palermo, Italy; b Department of Biological, Chemical and Pharmaceutical Sciences and Technologies (STEBICEF), University of Palermo, Italy; c Department of Neurobiology, Care Sciences and Society, Division of Neurogeriatrics, Center for Alzheimer Research, Karolinska Institutet, Stockholm, Sweden; d Euro Mediterranean Institute of Science and Technology-I.E.ME.S.T., Palermo, 90139, Italy

**Keywords:** Cognitive dysfunction, Redox homeostasis, Leptin signalling, Causal modelling, Anxiety-like behaviour

## Abstract

Metabolic dysfunction severely affects brain physiology; however, the progression of cognitive and affective alterations and their causal relationship with systemic dysmetabolism driving metabolic syndrome (MetS) have to be fully elucidated. Here, we addressed this hypothesis by combining longitudinal experimental data with a causal statistical modelling framework to explore mechanistic dependencies between cognitive and metabolic processes. To this aim, we used a 20-week high-fat diet (HFD) rat model of MetS, integrating assessment of anxiety-like behaviour, reactivity, and declarative memory with profiling of systemic metabolic, neuroendocrine and redox markers, as candidate neurometabolic mediators. Prolonged HFD exposure induced, together with an early and progressive metabolic dysregulation, a deterioration of anxiety-like behaviour and memory performance with specific temporal dynamics across behavioural domains. Our findings indicate that cognitive impairment is embedded within the progression of MetS, contributing to the organization and expansion of the neuro-metabolic phenotype. Furthermore, our multivariate analyses showed coordinated neurometabolic cascades with covariation of cognitive dysfunction with altered metabolic burden, oxidative stress, leptin signalling, and ketone body regulation. Importantly, causal modelling identified distinct neurometabolic pathways underlying domain-specific vulnerability. In particular, systemic leptin signalling emerged as an integrative signal linking metabolic load and neuroendocrine dysregulation with affective dimension, whereas memory impairment was preferentially linked to redox imbalance. Collectively, this study allows reconceptualization of MetS identifying cognitive–metabolic signatures and their causal architecture that thus provide a translational framework to interpret vulnerability profiles characterized by maladaptive behavioural regulation, with potential implications for early stratification and targeted intervention strategies.

## Highlights

• Cognitive dysfunction represents a mechanistic driver of metabolic syndrome progression.
• Causal statistical model reconstructs neurometabolic pathways implicated in behavioural vulnerability in specific cognitive domains.
• Leptin signalling mediates metabolic effects on affective dysfunction.
• Oxidative stress links metabolic dysfunction to memory impairment.

## Graphical abstract

**Figure:**
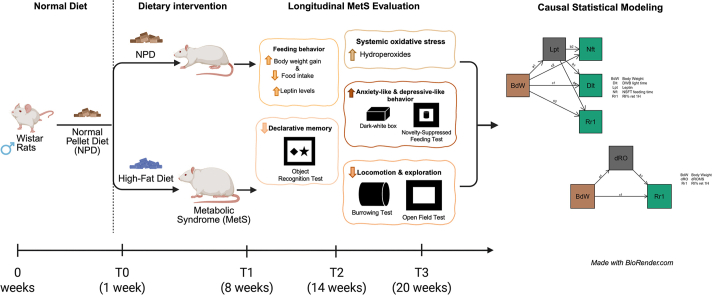

## 1 Introduction

Metabolic syndrome (MetS) is widely recognised for its peripheral metabolic consequences, while growing evidence suggests that its clinical condition can significantly compromise the central nervous system (Dinel et al., 2011; Zouridis et al., 2025). Cognitive dysfunction has been increasingly recognised as a relevant component of metabolic disorders, with impairments in cognitive flexibility, memory, and affective regulation, that could represent a transdiagnostic vulnerability factor predicting relapse and poor treatment outcomes (Chen et al., 2025; Gambino et al., 2020; Ray et al., 2017; Vagena et al., 2019). Cognitive impairment has indeed been associated with MetS, driven by converging mechanisms such as inflammation, oxidative stress and dysregulated neurotrophic signalling (Carrier, 2017; Cen et al., 2024; Dinel et al., 2011). However, the mechanistic basis linking systemic metabolic states to such global cognitive vulnerability remains poorly understood. These alterations may not merely represent downstream consequences of metabolic dysregulation, but they may constitute integral components of MetS pathophysiology, potentially disrupting brain homeostasis and influencing systemic metabolic control (Zheng et al., 2023). Individual metabolic abnormalities have been linked to specific aspects of brain dysfunction, without a comprehensive and mechanistic perspective accounting the neurometabolic marker's role in shaping MetS-associated dysfunctions (Diaz et al., 2018; Lv et al., 2024; Song et al., 2022). In this context, systemic oxy-inflammatory, dyslipidemic, dysglycemic and adipokine markers have emerged as key mediators associated with accelerated memory decline, affective regulation, global cognitive impairment, and reduced frontal and limbic cortical function (Alfaro et al., 2016; Atti et al., 2019; Brownlee, 2005; Cen et al., 2024; Dantoine et al., 2002; Luzzi et al., 2010; Siervo et al., 2011). Among these, leptin represents a prototypical adipokine at the interface between metabolic, neuroendocrine, and inflammatory signalling, playing a central role in feeding behaviour control, energy homeostasis and brain-metabolism communication. Altered leptin signalling and resistance have been associated with affective and cognitive dysfunction in MetS, potentially through dysregulation of the hypothalamic–pituitary–adrenal axis and the amplification of pro-inflammatory cytokine production (Chirinos et al., 2013; Di Majo et al., 2024a; Farr et al., 2015; Lucassen and Cizza, 2012; Morrison, 2009; Wozniak et al., 2009). In parallel, circulating ketone bodies such as β-hydroxybutyrate have recently been proposed as markers of impaired cerebral glucose utilization in MetS, with elevated levels reflecting a compensatory metabolic response rather than a neuroprotective mechanism, and implicated in cognitive decline (Gutierrez-Tordera et al., 2024). Yet, the neurophysiological mechanisms linking MetS to cognitive dysfunctional domains remain elusive since most rodent studies have concentrated on hippocampal-dependent performance, thus overlooking cortical and network-level time-dependent processes, characterized by progressive neuro-metabolic deterioration, sustained oxy-inflammation and dysregulated neurotransmission (Gambino et al., 2022; Lv et al., 2024; Song et al., 2022).

To fill this void, rodent models of High-Fat Diet (HFD) have been chosen to investigate MetS-associated cognitive dysfunction in a compressed time frame, since they facilitate the identification of systemic biomarkers linked to specific cognitive impairments. HFD feeding for 10 weeks can already trigger key pathological mechanisms, i.e. hippocampal inflammation, mitochondrial dysfunction, oxidative stress, and microglial hyperactivity, fostering neurodegeneration associated with increased activation of cortical necroptotic pathways and inflammasomes, as well as epigenetic changes that alter the expression of hippocampal microRNAs, thus highlighting the complex molecular pathways linking HFD and cognitive impairment (Ito et al., 2024; Keshk et al., 2020; Spinelli et al., 2024). Emerging evidence further indicates mitochondrial alterations as interrelated with persistent oxidative stress and activation of endoplasmic reticulum stress pathways, leading to a maladaptive feed-forward loop triggering neuroinflammatory signalling and progressive cognitive decline (de Mello et al., 2019; Dentoni et al., 2022). Metabolic hallmarks in HFD indeed were proven to correlate with oxidative stress, dysregulated neurotrophic signalling and behavioural impairments such as anxiety and memory deficits, as emerged by previous evidence highlighting the multifactorial impact of HFD on brain health (Di Majo et al., 2022, 2024a; Gambino et al., 2024; Wang et al., 2018; Woodruff et al., 2024; Xia et al., 2021; Xiong et al., 2022). Complementary omic approaches, ranging from unbiased hypothalamic proteomics (Evans et al., 2024) to metabolomics and microbiome profiling (Li et al., 2023; Loh et al., 2024), have further highlighted early metabolic rewiring, synaptic vulnerability, and neuroimmune priming as drivers of cognitive decline in HFD.

Despite substantial evidence supporting a progressive worsening of neuroinflammatory and neurodegenerative processes with prolonged HFD exposure (Saiyasit et al., 2020), a comprehensive, longitudinal definition of the putative cognitive phenotype associated with MetS progression is still lacking, as well as, a clear mechanistic understanding of how cognitive impairment evolves in the context of systemic metabolic deterioration and sustained oxy-inflammatory burden. Our aim is to test the hypothesis that cognitive dysfunction represents a mechanistically active component of disease progression, rather than a downstream consequence of metabolic impairment. Specifically, we sought to determine whether alterations in cognitive and affective domains track, anticipate, and potentially contribute to the longitudinal evolution of MetS, by using a prolonged 20-week HFD rat model. To address this question, we adopted an integrative framework based on causal statistical modelling, multivariate analysis and principal component analysis, designed to explore the mechanistic and directional relationship between behavioural profiling and systemic metabolic and redox markers. Circulating leptin, ketone bodies and redox markers were assessed as representative neurometabolic signals. Cognitive impairment was assessed across multiple domains including exploratory behaviour, stress reactivity, affective state and declarative memory allowing the construction of a multidimensional behavioural phenotype across time.

This approach allowed us to capture how early metabolic propagation through neuroendocrine and oxidative pathways led to the gradual emergence and consolidation of cognitive and affective dysfunction, providing a dynamic view of MetS as an expanding neuro-metabolic phenotype. In this framework, MetS could be conceptualized as a condition in which systemic metabolic dysregulation is tightly associated with cognitive vulnerability, reflecting integrated alterations in metabolic and brain function.

## 2 Materials and methods

### 2.1 Animals

Male Wistar rats were supplied by Envigo S. r.l (Indianapolis, Indiana), housed two per cage and sustained on a 12-h light/dark cycle at a constant temperature (22–24 ◦C) and humidity (50 ± 10%). Throughout the seven-days acclimatation phase, animals were manipulated and initially nourished with a standard chow diet and then separated into two uniform groups with balanced weight (7-week-old, n = 12, weighing 240 ± 13.04 g). The control group (NPD: normal pelletized diet) received a low-fat regimen consisting of a standard rat chow (3.94 kcal/g) (code PF1609, certificate EN 4RF25, Mucedola, Milan, Italy). The second group (HFD: high-fat diet) was nourished for 20 weeks with a hypercaloric pelletized diet (5.5 kcal/g), with 60% of energy sourced from fats (code PF4215-PELLET, Mucedola, Milan, Italy), consisting of 34% lipid, 23% protein, 38% carbohydrate and 5% fiber (Pipitone et al., 2023). All animals had unrestricted access to food and water. During the experimental protocol, animal care and handling adhered to the ARRIVE guidelines 2.0 and the European Directive (2010/63/EU). The experimental procedures received approval from the animal welfare committee of the University of Palermo and were authorized by the Ministry of Health (Rome, Italy; Authorization Number 386/2024-PR).

### 2.2 Experimental design

The experimental study is segmented into four distinct phases:

- Initial group allocation (T0): after acclimatization, animals were fed with HFD for 8 weeks until the induction of MetS;
- MetS induction (T1): verified at T1 after 8 weeks of HFD, employing established protocols (Gambino et al., 2023; Rodríguez-Correa et al., 2020);
- MetS progression (T2): evaluated at 14 weeks to monitor metabolic and behavioural parameters;
- Experiment conclusion (T3): at 20 weeks after T0 animals were sacrificed, following authorized procedures.

### 2.3 Determination of metabolic syndrome

In order to identify the induction of MetS, based on established criteria (Rodríguez-Correa et al., 2020), after 8 weeks of diet, body weight gain was evaluated together with biochemical parameters from representative plasma samples including triglycerides (TG) and glucose tolerance (GTT). Once differences were identified between HFD and NPD groups, further analyses were performed on rats in the two groups at “T1” time, defined as the stage of metabolic syndrome onset.

### 2.4 Biometric and metabolic parameters

Throughout the study, MetS systemic alterations were assessed focusing on changes in biometric, biochemical, and oxidative homeostasis parameters. Plasma samples were collected at all timepoints for biochemical analyses to assess metabolic status and oxidative stress biomarkers. Samples failing to meet predefined analytical quality criteria (e.g. hemolysis or low yield) were excluded from the analysis.

#### 2.4.1 Biometric parameters: body weight gain, food and water intake

Rats were weighed weekly, together with their food intake that was assessed as the difference between the weight of the chow provided and the remaining chow. Additionally, water intake was monitored weekly using a 300 mL water bottle by calculating the difference between the initial weight of the full bottle and the weight of the remaining water after exposure.

#### 2.4.2 Evaluation of leptin levels

Analysis of plasma leptin levels was performed by enzyme immunoassay using the Quantikine ELISA kit from R&D System, Bio-Techne, 614 McKinley Place NE, Minneapolis, MN 55413 USA. Sample dilution factors were determined based on the absorbance values obtained from an initial 1:2 dilution, as per the manufacturer's protocol, to ensure all readings fell within the linearity range of the standard curve. Plasma leptin concentrations (pg/mL) were determined by interpolating absorbance readings from a mean standard curve (R^2^ = 0,98) covering a range of 31.3 to 2000 pg/mL. Absorbance was measured using a UV/Vis microplate reader at 450 nm, with wavelength correction set at 540 nm.

#### 2.4.3 Lipid homeostasis

Lipid homeostasis in terms of triglycerides (TG) and total cholesterol levels concentrations was measured in plasma samples using commercially available kits employing the Free Carpe Diem device (FREE® Carpe Diem; Diacron International, Grosseto, Italy), as in (Di Majo et al., 2022). Data are presented in mg/dL.

#### 2.4.4 Evaluation of ketone levels

After an overnight fast, a drop of blood was collected from the tail vein of the rat and applied on a blood ketone test strip to measure a baseline β-ketone (beta-hydroxybutyrate) value using a Ketone meter (KetoBM). The measurement range of β-ketone concentration in capillary whole blood is 0.0 to 8.0 mmol/L.

#### 2.4.5 Glucose tolerance

Glucose Tolerance Test (GTT), a widely used diagnostic tool to evaluate insulin resistance and glucose dysmetabolism, was performed according to established procedures (Bowe et al., 2014; Di Majo et al., 2022).

### 2.5 Oxidative stress parameters

Plasma oxidative stress was assessed using Diacron kits, as in (Di Majo et al., 2022; Gambino et al., 2023). Analyses were performed on plasma samples using the FREE® Carpe Diem device (Diacron International, Grosseto, Italy). The prooxidant status was evaluated through the dROM test and the LP-CHOLOX assay. The dROM test quantifies hydroperoxides in Carratelli units (UCARR, standard range is 250–300 U. CARR with 1 U. CARR corresponding to 0.08 mg/dL of H_2_O_2_) (Lee et al., 2017). Lipid peroxidation and oxidized cholesterol levels were determined using LP-CHOLOX, based on peroxides capacity to catalyse Fe^2+^ oxidation and spectrophotometric detection at 505 nm (Macri et al., 2015; Mancini et al., 2017). The results are reported in mEq/L.

### 2.6 Behavioural assessment

All the protocols, except for burrowing, were performed via a behavioural tracking software at T1, T2 and T3 (AnyMaze, Version 7.49) and analysed by a blinded trained scientist.

#### 2.6.1 Exploratory behaviour and reactivity

##### 2.6.1.1 Burrowing test

The burrowing test was performed as previously reported (Koska et al., 2019). Plastic burrowing tubes (32 cm long x 10 cm Ø, elevated on one site by 6 cm) were filled with 2.3 kg gravel (quartz-light, grain size 2 - 4 mm) and positioned within the cage. Each rat underwent testing individually. All rats underwent a training session between T0 and T1: on day 1, rats were provided with an empty burrowing tube for 30 min; on day 2 rats were given a gravel-filled tube for 30 min; on day 3-4 rats were given a gravel-filled tube for 60 min. Burrowing behaviour was then assessed at all time points, evaluating the latency period before initiating burrowing and the amount of burrowed gravel by the difference between the remaining gravel and the initial weight in the tube. Animals that did not initiate burrowing within 60 min were excluded from the statistical analysis, as previously (Di Liberto et al., 2018).

##### 2.6.1.2 Open field test (OFT)

The open field test (OFT) was employed to assess locomotor and exploratory behaviour (Seibenhener and Wooten, 2015). Subjects were introduced into a square open field (70 × 70cm) positioned facing the wall from a distance of 10 cm and were subsequently recorded for a duration of 10 min. Recorded parameters included total distance covered, immobility time, time spent in the centre zone and frequency of entries in the zone (Gambino et al., 2024).

#### 2.6.2 Anxiety-associated behaviour

##### 2.6.2.1 Novelty-suppressed feeding test (NSFT)

In the novelty-suppressed feeding test (NSFT), following a 24-h period of food deprivation, rats were introduced in an open-field maze where a food pellet (2 g of HFD or NPD chow) was positioned on a white rounded disc at the centre of the arena. Animals had a 10-min cut-off to reach and consume the food pellet, only rats that completed the test were included in the analysis (Blasco-Serra et al., 2017). The feeding duration was monitored. Once rats completed the test, they were promptly removed from the arena and returned to their home cage, where the amount of food consumed by animals in the subsequent 10 min was measured to confirm deprived conditions.

##### 2.6.2.2 Dark-white box test (DWB)

For the dark-white box test (DWB), a plastic box (60 cm length × 30 cm height × 30 cm width) partitioned into two equal-size compartments connected through a dimly lighted tunnel was used. One compartment was painted black and covered with a lid, the other compartment was painted white and illuminated with a 45 W LED light bulb (3000-4000 lumens) positioned 40 cm above the box (Frinchi et al., 2020). The animals were placed in the white compartment facing the tunnel and, for 5 min, the number of entries and the time spent in the light zone were evaluated.

#### 2.6.3 Learning and memory: object recognition test

The Object recognition test (ORT) is recognised as a reliable and widely employed method for evaluating the declarative memory system in experimental models (Gambino et al., 2024; Squire and Zola, 1996). After habituation to the open field arena (same as in 2.5.1.2), animals were presented with two identical objects placed at a fixed distance from each other. Following a 1-h retention period, one of the two objects was replaced with a novel one, while the other remained unchanged (“familiar object”). After 24 h, the familiar object and a further new object, different from the previous one, were placed inside the arena. The objects were selected and 3D printed based on specific literature pertaining to this paradigm (Inayat et al., 2021). We considered the retention index (RI%), representing the time spent exploring the novel object relative to the total time spent investigating both the familiar and novel one, as an animal's ability to discriminate between a novel and a familiar stimulus (Botton et al., 2010).

### 2.7 Statistical analyses and causal modelling

Statistical analysis was conducted using GraphPad Prism 9.02 (San Diego, CA, USA). Biometric, biochemical and behavioural parameters were compared using a two-way repeated measures (RM) or mixed-effects ANOVA model, as appropriate, followed by Bonferroni post hoc test for significant differences in both within- and between-subject comparisons, to assess the effects of “time”, “diet” and their interaction. Statistical significance was established at *p* < 0.05. Additionally, the statistical power (g-power) was considered if > 0.75 and the effect size was evaluated if > 0.40. The results are presented as the mean ± standard deviation (SD). Complementary analyses were performed using custom scripts in MATLAB 2025B (The MathWorks, Inc.) to characterize the evolution of the cognitive and metabolic phenotype. To visualize the progressive expansion of the pathological phenotype, comparative spider plots were generated. Data were normalized using Z-scores calculated across the entire dataset (NPD and HFD combined). The directionality of performance indices (e.g., Recognition Index) was inverted so that outward expansion in the plot consistently represented phenotypic deterioration. Furthermore, to inquire the relationship between metabolic and cognitive markers Principal Component Analysis and causal statistical model (Zhu et al., 2022) were performed in JASP. All variables were z-score standardized for scale independency. For PCA Factor extraction is based on Kaised criterion (eigenvalue >1.0) with visual inspection of the scree plot. Component rotation was applied using Promax rotation with Kaiser normalization. Model fit was assessed via Chi-squared goodness-of-fit testing. A mediation model was estimated in JASP with Body Weight (BdW) as the predictor, Leptin (Lpt) as the mediator, and three outcomes: DWB Light Time (Dlt), NSFT feeding time (Nft), and RI% ret 1H (Rr1). Additionally, a separate mediation model tested dROM (systemic oxidative stress) as an alternative mediator linking Body Weight to RI% ret 1H memory performance. All models were estimated with mean-centered variables and included direct paths (BdW→outcomes), mediator paths (BdW→mediator), outcome paths (mediator→outcomes), and both indirect (mediated) and total effects with 95% confidence intervals.

## 3 Results

### 3.1 Effects of HFD on body weight, food and water intake, and leptin levels

**Fig. 1:**
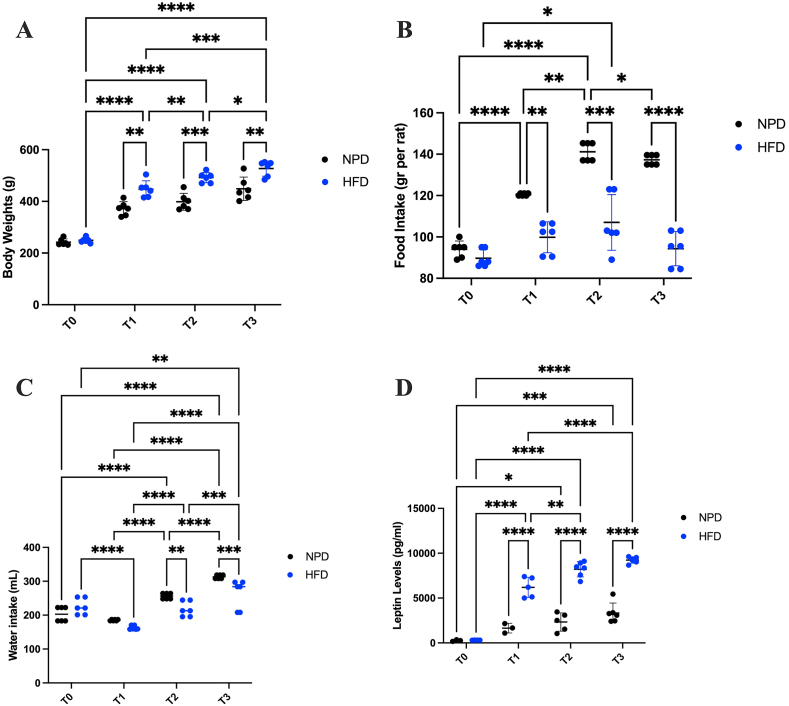
Biometric parameters and Leptin levels in NPD and HFD groups at T0, T1, T2 and T3. **A.** Variation of body weight gain (ΔBW). NPD and HFD at all time points n = 6. **B.** Average of food intake consumption (g per cage). NPD and HFD at all time points n = 6. **C.** Average of water intake consumption (mL per cage). NPD and HFD at all time points n = 6. **D.** Systemic Leptin levels. NPD at T0 n = 3; NPD at T1 n = 3; NPD at T2 n = 5; NPD at T3 n = 6; HFD at T0 n = 4; HFD at T1 n = 5; HFD at T2 n = 6; HFD at T3 n = 6. Statistical significance by two-way ANOVA followed by Bonferroni post hoc tests is indicated by (∗)p < 0.05, (∗∗)p < 0.001, (∗∗∗)p < 0.0001 and (∗∗∗∗)p < 0.0001.

Body weight, food intake and water intake were monitored throughout the experimental period in the NPD and HFD groups. Firstly, a two-way ANOVA conducted on body weight revealed significant main effects of time (F_(1.708,17.08)_ = 638.6, p < 0.0001) and dietary treatment (F_(1,10)_ = 18.80, p = 0.0015), as well as a significant interaction between these factors (F_(3,30)_ = 21.24, p < 0.0001) (Fig. 1A). Post-hoc assay showed no significant differences among groups at baseline (T0), confirming comparable initial conditions. After 8 weeks (T1), body weight was significantly increased in the HFD group compared with NPD control, and this difference remains evident at both T2 and T3, progressively worsening within the HFD-group. As for food intake, two-way ANOVA showed significant effects of time (F_(1.319,13.19)_ = 38.51, p < 0.0001), nutritional treatment (F_(1,10)_ = 938.5, p < 0.0001) and their interaction (F_(3,30)_ = 14.61, p < 0.0001) (Fig. 1B). At baseline (T0), food consumption did not differ between groups. From T1 onwards, HFD groups reduced their food intake more than NPD controls. Similarly, while NPD animals displayed a gradual increase in water consumption across time, water intake was first significantly reduced within the HFD group at T1 versus T0, and then increased at T2 and T3. As for between-subjects comparison, the HFD group showed lower water intake than NPD at T2 and T3. Two-way ANOVA confirmed a significant difference in time (F_(3,30)_ = 99.90, p < 0.0001), nutritional treatment (F_(1,10)_ = 6.308, p = 0.0308) and their interaction (F_(3,30)_ = 11.11, p < 0.0001) (Fig. 1C). Finally, systemic concentrations of leptin were measured in all experimental groups by ELISA assay. Two-way ANOVA demonstrated a significant difference in time (F_(3,19)_ = 112.2, p < 0.0001), nutritional treatment (F_(1,11)_ = 110.2, p < 0.0001) and their interaction (F_(3,19)_ = 35.80, p < 0.0001). Bonferroni post-hoc test showed that leptin levels were the same in both groups at baseline and started to increase at T1. In particular, the HFD group had increased leptin levels compared to NPD and within-group over time (Fig. 1D).

### 3.2 Effects of HFD on metabolic and systemic oxidative stress parameters

**Fig. 2:**
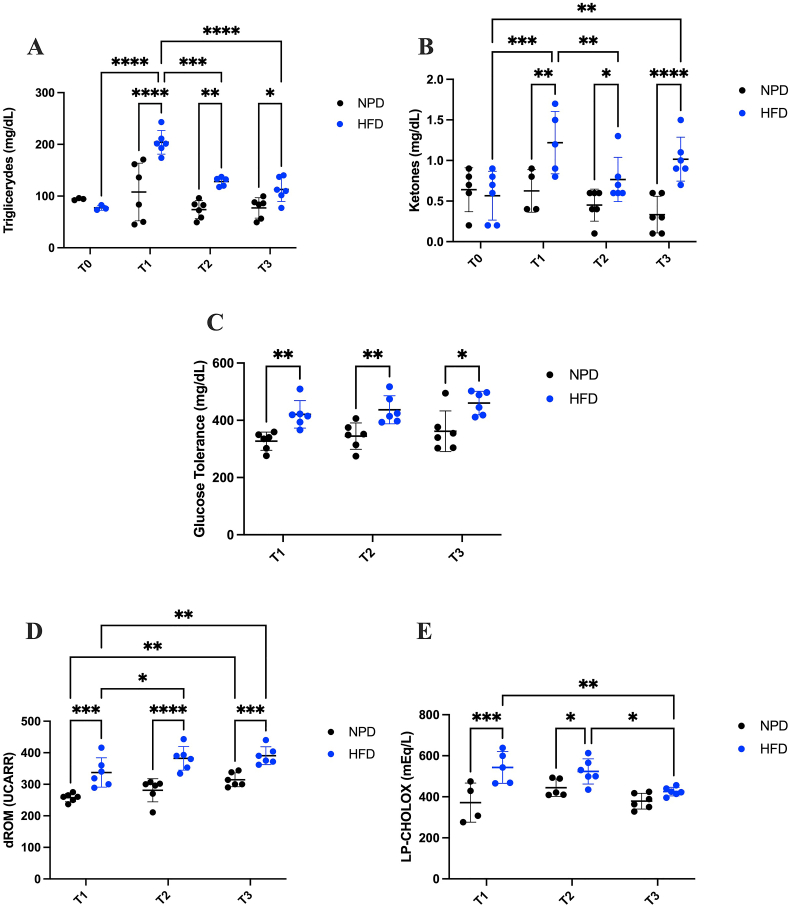
Metabolic and Prooxidant status biomarkers between HFD and NPD experimental groups at T0, T1, T2 and T3. **A.** Triglycerides (TG) levels. NPD at T0 n = 3; NPD at T1 n = 6; NPD at T2 n = 6; NPD at T3 n = 6; HFD at T0 n = 3; HFD at T1 n = 6; HFD at T2 n = 5; HFD at T3 n = 6. **B.** Ketone levels. NPD at T0 n = 5; NPD at T1 n = 4; NPD at T2 n = 6; NPD at T3 n = 6; HFD at T0 n = 6; HFD at T1 n = 5; HFD at T2 n = 6; HFD at T3 n = 6. **C.** Glucose Homeostasis (mg/dL). Plasma glucose levels (mg/dL) per unit of time (h). NPD and HFD at all time points n = 6. **D.** dROM (UCARR). NPD and HFD at all time points n = 6. **E.** LP-CHOLOX (mEq/L). NPD at T1 n = 4; NPD at T2 n = 5; NPD at T3 n = 6; HFD at T1 n = 5; HFD at T2 n = 6; HFD at T3 n = 6. Statistical significance by two-way ANOVA followed by Bonferroni post hoc tests is indicated by (∗)p < 0.05, (∗∗)p < 0.01, (∗∗∗)p < 0.0001 and (∗∗∗∗)p < 0.0001, as represented in the graphs.

The lipid profile, ketone levels and glucose tolerance were evaluated between the HFD and NPD groups throughout the experimental period. Two-way ANOVA performed on the plasma concentration of TG pointed out differences between groups in time (F_(3,23)_ = 14.64, p < 0.0001), nutritional treatment (F_(1,10)_ = 21.44, p = 0.0009) and their interaction (F_(3,23)_ = 6.172, p = 0.0031). Post-hoc analyses showed a stable significant increase in HFD versus NPD over time, reaching a peak at T1 and then reducing at later timepoints (Fig. 2A). Statistical analysis performed on the total cholesterol levels revealed significant increases in HFD vs NPD, though not reaching significance at all timepoints and experimental conditions, as assessed by two-way anova (Supplementary Figure 1). Similarly, statistical evaluation of ketone levels by two-way ANOVA, followed by Bonferroni post-hoc analysis, revealed significant effects of time (F_(3,25)_ = 5.672, p = 0.0042), nutritional treatment (F_(1,11)_ = 10.06, p = 0.0089) and their interaction (F_(3,25)_ = 7.443, p = 0.0010, Fig. 2B). In detail, HFD groups displayed steadily higher ketone levels compared to NPD controls throughout the experimental period, with a dynamic temporal pattern characterized by an early peak at T1, a transient decrease, and a later recovery. Moreover, glucose homeostasis evaluated at 8th, 14th and 20th weeks outlined significant differences in glucose tolerance between the experimental groups NPD and HFD, as assessed by the AUC (area under the curve) via GTT. Two-way ANOVA showed a significant increase in AUC values in the HFD group compared to the NPD control, showing significant difference in nutritional treatment (F_(1,10)_ = 24.11, p = 0.0006) but not in time (F_(1.889,18.89)_ = 2.125, p = 0.1490) and their interaction (F_(2,20)_ = 0.01799,p = 0.9822, Fig. 2C). Furthermore, plasma samples collected from all experimental groups were analysed to evaluate the prooxidant status, thus assessing systemic oxidative stress associated with MetS. In particular, dROM levels differed significantly across the experimental groups over time (F_(2,20)_ = 13.51, p = 0.0002) and according to nutritional treatment (F_(1,10)_ = 36.10, p = 0.0001), while their interaction has not shown significant difference (F_(2,20)_ = 0.8126, p = 0.4578). The post hoc analysis highlighted a pronounced increase of dROM production in the HFD rats vs NPD, that significantly worsened from T1 to T3 (Fig. 2D). Additionally, LP-CHOLOX levels were significantly elevated in the HFD group. Two-way ANOVA revealed significant effects in time (F_(2,16)_ = 6.456, p = 0.0088), nutritional treatment (F_(1,10)_ = 20.53, p = 0.0011), and a tendency towards significance in their interaction (F_(2,16)_ = 3.169, p = 0.0693) (Fig. 2E). Post-hoc analysis revealed higher levels of LP-CHOLOX in HFD vs NPD at T1 and T2.

### 3.3 Effects of HFD on behavioural parameters

**Fig. 3:**
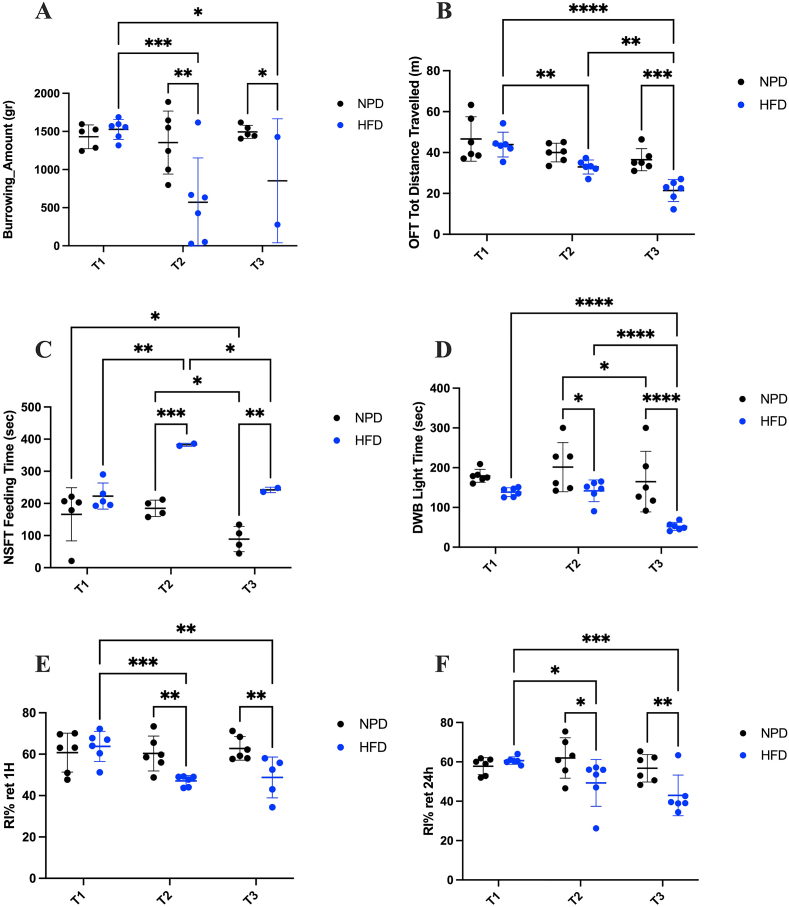
Behavioural parameters assessed in the Open Field, Burrowing, Novelty-Suppressed Feeding (NSFT), Dark-White Box (DWB) and Object Recognition tests in NPD and HFD groups at T1, T2 and T3. **A.** Amount of gravel burrowed (gr). NPD at T1 n = 5; NPD at T2 n = 6; NPD at T3 n = 5; HFD at T1 n = 6; HFD at T2 n = 6; HFD at T3 n = 2. **B.** Total distance travelled (m). NPD and HFD at all time points n = 6. **C.** Feeding time (sec). NPD at T1 n = 5; NPD at T2 n = 4; NPD at T3 n = 4; HFD at T1 n = 5; HFD at T2 n = 2; HFD at T3 n = 2. **D.** Time spent in the light area. NPD and HFD at all time points n = 6. **E.** (RI%) after 1-H of retention (ret 1 h). NPD at T1 n = 6; NPD at T2 n = 6; NPD at T3 n = 6; HFD at T1 n = 6; HFD at T2 n = 6; HFD at T3 n = 5. **F.** (RI%) after 24-H retention (ret 24 h). NPD and HFD at all time points n = 6. Statistical significance by two-way ANOVA followed by Bonferroni post hoc tests is indicated by (∗)p < 0.05, (∗∗)p < 0.001, (∗∗∗)p < 0.0001 and (∗∗∗∗)p*<*0.0001.

Animals were assessed in the burrowing and OFT to evaluate their reactivity and exploratory behaviour across the experimental period. The analysis of burrowing amount revealed significant main effects of time (F_(2,14)_ = 6.870, p = 0.0083), nutritional treatment (F_(1,10)_ = 8.343, p = 0.0161), and their interaction (F_(2,14)_ = 5.179, p = 0.0207), as determined by two-way ANOVA followed by Bonferroni post hoc test. These data showed that HFD displaced significantly less gravel compared to NPD controls and this reduction further worsened over time within the HFD group (Fig. 3A). With respect to burrowing latency, two-way ANOVA did not reveal a significant difference between groups, though HFD showed a tendency to start burrowing later than NPD (Supplementary Figure 2). In the total distance travelled in OFT, two-way ANOVA revealed significant differences in time (F_(2,20)_ = 23.57, p < 0.0001), nutritional treatment (F_(1,10)_ = 11.32, p = 0.0072) and their interaction (F_(2,20)_ = 3.503, p = 0.0496). Bonferroni post-hoc test showed that total distance progressively decreased over time in HFD from T2, which further worsened at T3 compared to NPD (Fig. 3B). As for time spent at the centre of the open field maze, HFD rats displayed a decreasing trend over time compared to NPD controls, although no statistical significance is observed via two-way ANOVA in terms of time (F_(2,18)_ = 36.42, p < 0.0001), nutritional treatment (F_(1,10)_ = 22.57, p = 0.0008) and their interaction (F_(2,18)_ = 0.2372, p = 0.7913) (Supplementary Figure 3).

As for anxiety-like behaviour, the NSFT and DWB revealed differences between the two experimental groups. As for NSFT feeding time, two-way ANOVA followed by Bonferroni post hoc test showed significant effect on time (F_(2,7)_ = 10.96, p = 0.0070), nutritional treatment (F_(1,9)_ = 11.32, p = 0.0007) and their interaction (F_(2,7)_ = 4.749, p = 0.0498). This analysis revealed a significant increase in feeding time in the HFD group compared to NPD, particularly at T2 (Fig. 3C). Additionally, the food intake measured in the home cage in the 10 min following the test did not differ significantly between HFD and NPD groups (Supplementary Figure 4). As for time spent in the light zone in the DWB, two-way ANOVA evidenced a significant main effect of time (F_(2,20)_ = 16.03, p < 0.0001), nutritional treatment (F_(1,10)_ = 12.07, p = 0.0060) and their interaction (F_(2,20)_ = 4.984, p = 0.0175). In detail, HFD animals showed a significant reduction in light time from T2 onwards, which further worsened at T3. Light time was also significantly lower in the HFD group compared to NPD controls at T2 and T3. (Fig. 3D). Similarly, two-way ANOVA conducted on number of entries in the light zone showed significance differences in time (F_(2,28)_ = 10.34, p = 0.0004), nutritional treatment (F_(1,28)_ = 10.10, p = 0.0036) and their interaction (F_(2,28)_ = 5.401, p = 0.0104) by Bonferroni post-hoc test, revealing a significant reduction within HFD animals over time and also with respect to NPD at T3 (Supplementary Figure 5).

The effect of HFD on declarative memory was evaluated in the ORT. During training with two identical objects, no significant difference in RI% was observed across the experimental groups, with the index remaining consistently around 50% (Supplementary Figure 6). After 1-h retention, two-way ANOVA followed by Bonferroni post-hoc test showed in time (F_(2,19)_ = 4.758, p = 0.0211), nutritional treatment (F_(1,10)_ = 7.959, p = 0.0181) and their interaction (F_(2,19)_ = 5.430, p = 0.0136). Post hoc analysis showed a significantly lower RI% in HFD rats versus NPD at T2 and T3, as well as a progressive reduction across T1-T3 (Fig. 3E). Only one HFD animal at T3 did not explore any objects and was therefore excluded from analysis. Similarly, at 24-h retention, two-way ANOVA showed a significantly lower RI% in HFD group across time and compared to NPD control, revealing a significant difference in time (F_(2,20)_ = 5.201, p = 0.0152), nutritional treatment (F_(1,10)_ = 5.057, p = 0.0483) and their interaction (F_(2,20)_ = 5.050, p = 0.0168) (Fig. 3F). Indeed, HFD rats did not manage to recognize the novel stimulus and spent more time exploring the familiar one (RI%<50%) with respect to NPD (p < 0.05).

### 3.4 Causal modelling of biometric, behavioural and biochemical parameters

#### 3.4.1 Phenotype expansion

**Fig. 4:**
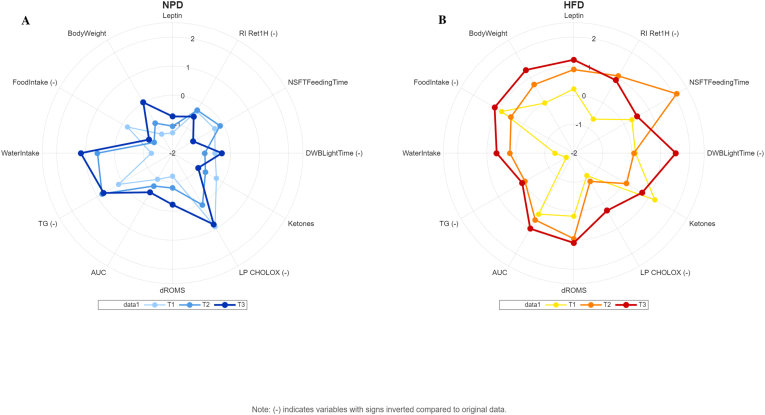
Spatio-temporal evolution of the neuro-metabolic phenotype. Radar charts illustrating the multivariate trajectory of NPD (**A**) and HFD (**B**) groups across three timepoints (T1, T2, T3). Variables were Z-score normalized and aligned such that outward expansion represents a worsening of the phenotype (pathological shift). While NPD animals maintain a stable profile, HFDs exhibit a progressive expansion, highlighting the deterioration of metabolic markers (Leptin, Ketones, Body Weight) and cognitive/behavioural performance (RI, DWB, NSFT).

To integrate metabolic and neurocognitive alterations, the phenotypic evolution of NPD and HFD groups was mapped over time (Fig. 4). The NPD group (Fig. 4A) maintained a stable, homeostatic profile throughout T1–T3. Minor fluctuations are observed without systemic expansion. In normal diet physiological homeostasis is maintained across neuro-metabolic domains. In contrast, the HFD group (Fig. 4B) displayed time-dependent, progressive deterioration, with a phenotype expansion. The polygon area increases systematically from T1 to T3. In T1 an initial expansion is related predominantly to Leptin, Body Weight and Ketones, cognitive markers show modest outward shift compared to NPD. In T3, the maximal phenotype expansion across all seven dimensions.

**Fig. 5:**
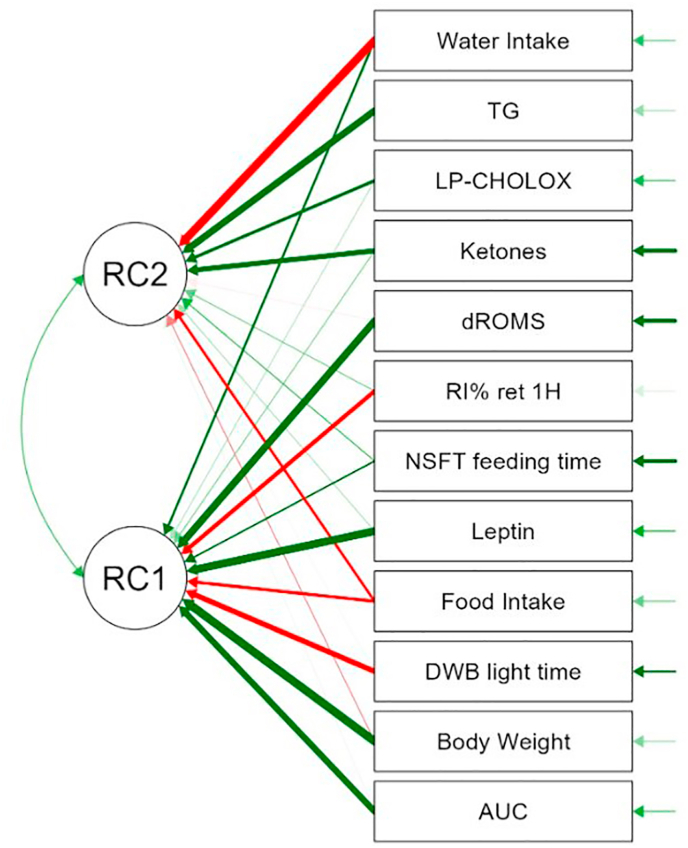
Two-dimensional path diagram illustrating the loading structure of RC1 and RC2 from rotated Principal Component Analysis. Factors are displayed as rectangular nodes with directional paths indicating component loadings. Green lines denote positive loadings; red lines denote negative loadings. Line thickness is proportional to loading magnitude. (For interpretation of the references to colour in this figure legend, the reader is referred to the Web version of this article.)

#### 3.4.2 Principal component analysis

Results from PCA are shown in Fig. 5 and in Supplementary Tables 1A, 1B and 1C. Eigenvalue analysis revealed a two-component solution with robust statistical support [χ^2^(51) = 80.71, df = 43, p < 0.001] (Table 1A, *Supplementary***)**. In the unrotated solution, Component 1 (eigenvalue = 5.932) accounted for 45.6% of total variance, while Component 2 (eigenvalue = 2.517) contributed an additional 19.4%, yielding cumulative explained variance of 65.0% (Table 1C, *Supplementary*). Following Promax rotation with Kaiser normalization, the components exhibited moderate intercorrelation. RC1 emerged as the primary variance axis, characterized by strong positive loadings for metabolic markers (Body Weight r = 0.910; Leptin r = 0.860; dROM r = 0.919; AUC r = 0.616) and moderate negative loadings for cognitive-behavioural measures (DWB Light Time r = −0.768; RI% ret 1H r = −0.476). NSFT Feeding Time showed a moderate positive loading (r = 0.450). RC1 captures a cognitive dysfunction dimension scaling with metabolic burden. RC2 accounted for secondary variance, with strong positive loadings for lipid peroxidation markers (LP-CHOLOX r = 0.905) and moderate loadings for triglycerides (TG, r = 0.607), Ketones (r = 0.507), and NSFT Feeding Time (r = 0.507). Water Intake displayed a strong negative loading (r = −0.956), alongside Food Intake (r = −0.467) (Table 1B, *Supplementary* ).

#### 3.4.3 Causal statistical model

**Fig. 6:**
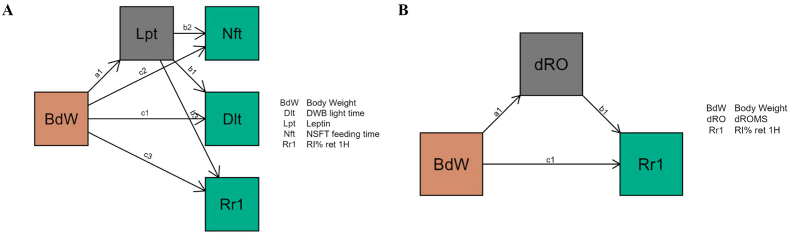
Representative Causal Statistical Models. **A.** Path model testing leptin-mediated associations between Body Weight (BdW) and anxiety-like behaviour (DWB light time; Dlt), novelty-suppressed feeding latency (NSFT feeding time; Nft), and object recognition memory performance (RI% ret 1 h; Rr1). **B.** Path model linking body weight, systemic oxidative stress, and memory performance. Path analysis tests whether reactive oxygen metabolites (dROMS) mediate the association between Body Weight (BdW) and object recognition memory (RI% ret 1 h; Rr1).

The causal statistical models are shown in Fig. 6. The mediation effects comprehend both the direct and indirect paths mediated by leptin and dROMS. The first mediation model includes direct paths from Body Weight to DWB light time, NSFT feeding time, and RI% ret 1H, as well as indirect paths via Leptin (Table 2, *Supplementary;* and Fig. 6A). Body weight positively predicted leptin (Table 2A, *Supplementary*). Leptin significantly predicted both anxiety-like outcomes, showing a negative association with DWB light time (Table 2A, *Supplementary*) and a positive association with NSFT feeding time (Table 2A, *Supplementary*). In contrast, leptin did not significantly predict RI% ret 1H (Table 2A, *Supplementary*). Direct effects of body weight on the outcomes were not significant (BdW→DWB light time; BdW→NSFT feeding time; BdW→RI% ret 1H; Table 2B, *Supplementary*). Indirect effects via leptin were significant for DWB light time and NSFT feeding time (Table 2B, *Supplementary*) but not for RI% ret 1H (Table 2B, S*upplementary*). Total effects indicated an overall negative effect of body weight on DWB light time (Table 2C, *Supplementary*) and an overall positive effect on NSFT feeding time (Table 2C, *Supplementary*). The total effect of body weight on RI% ret 1H was not significant (Table 2C, *Supplementary*). In the second path model, body weight significantly predicted dROMS (Fig. 6B and Table 3A, *Supplementary*), indicating that higher body weight was associated with increased systemic oxidative stress. The direct effect of body weight on RI% ret 1H was not significant (Table 3B *Supplementary*). dROMS showed a trend-level negative association with RI% ret 1H (Table 3A, *Supplementary*). The indirect effect of body weight on RI% ret 1H via dROMS was negative and close to significance (Table 3B, *Supplementary*), suggesting a potential mediation pattern. Despite the non-significant direct path, the total effect of body weight on RI% ret 1H was significant (Table 3C, *Supplementary*), indicating an overall association between greater body weight and poorer object recognition performance.

## 4 Discussion

Metabolic dysfunction severely affects brain physiology; however, the progression of cognitive and affective alterations and their causal relationship with systemic dysmetabolism driving obesity have to be fully elucidated (Gambino et al., 2020). The current study provides longitudinal, model-based evidence that chronic HFD exposure is associated with progressive cognitive and affective domain alterations, possibly through convergent metabolic and redox-related mechanisms. Rather than representing isolated domain-specific impairments, our data support a partially dissociable organization of cognitive and affective dysfunction, with distinct behavioural and metabolic correlates contributing to each domain.

To begin with, HFD rats were monitored for biometric and feeding-related parameters, such as body weight, food and water intake, and circulating leptin levels. An increase in body weight was found in HFD rats, that was significant not only in comparison with controls but also over time within the same group, indicating a progressive worsening of the obese phenotype. Such a pronounced increase in body weight was associated with a lower food intake than controls, that remained stable over time and likely reflects the higher caloric density of the diet. Consistent with the fact that excessive caloric consumption was not primarily driven by hyperphagic feeding behaviour per se, circulating leptin levels were persistently higher in HFD rats over time and compared with controls. This suggests an intact leptin-mediated suppression of food intake, but a failure of physiological leptin signalling to effectively restrain body weight gain. Water intake was reduced in HFD rats compared with controls without any significant change over time, suggesting that a hyperlipidic diet may induce adaptations in fluid and electrolyte homeostasis. In line with that, it was evidenced by an inverse relationship between water intake and obesity since higher fluid consumption is associated with reduced insulin resistance (Moosavi et al., 2025). Body water distribution in obesity results to be altered due to a lower proportion of total body water relative to body weight, increasing vulnerability to dehydration. This has been explained because of impaired thirst perception and reduced osmoreceptor sensitivity, which may delay dehydration recognition and fluid replacement (Crintea et al., 2025; Ganio et al., 2011; Sá et al., 2025). In addition, evidence revealed that dehydration is associated with disrupted emotional regulation and cognitive performance (Ganio et al., 2011). Together, these biometric outcomes point to a dysregulation of metabolic homeostasis, thus we subsequently assessed systemic lipid and glucose metabolic profiles. Plasma triglyceride and ketone bodies levels were maximal in HFD rats compared with NPD at T1, followed by a relative reduction at later time points. Such time-dependent dynamics do not account for an amelioration of dysmetabolism, though rather hints at a compensatory homeostatic response over time highly relying on ketone metabolism to counterbalance progressive lipid dysregulation. Therefore, the observed decrease at T2 and T3 likely reflects a new metabolic steady state rather than a reversal of dyslipidemia. As far as glucose homeostasis is concerned, HFD rats also displayed glucose intolerance that remained stable across time, suggesting that maximal impairment is reached early and maintained throughout disease progression.

All considered, such a transient increase in circulating ketone bodies at the onset of the MetS phenotype may reflect an early metabolic adaptation to impaired cerebral glucose utilization rather than a neuroprotective shift in energy metabolism (Fulghum et al., 2024; Prins, 2008). It was consistently reported that elevated plasma β-hydroxybutyrate levels are associated with poorer cognitive performance, accelerated cognitive decline, and increased risk of dementia, independent of diabetes status or APOE genotype (Chevli et al., 2024; Gutierrez-Tordera et al., 2024).

Given the key mechanistic role of oxidative stress in the pathogenesis of obesity and MetS, we next investigated well-established circulating biomarkers that provide complementary information on lipid peroxidation and hydroperoxide burden, as extensively validated in HFD models (Di Majo et al., 2022, 2024b; Gambino et al., 2023; Gasparro et al., 2024; Lasker et al., 2019). Oxidative stress was found not only to accompany but often to precede the onset of a HFD–induced insulin resistance and obesity, indicating its early and central role in metabolic dysregulation (Pieri et al., 2023), since nutrient oversupply and lipid accumulation promote reactive oxygen species (ROS) generation, mitochondrial dysfunction, and lipid peroxidation. In particular, we assessed systemic LP-CHOLOX levels, reflecting oxidized cholesterol-derived lipid peroxides. HFD rats showed increased values if compared with controls, confirming the presence of a sustained pro-oxidative milieu associated with diet-induced obesity. However, LP-CHOLOX levels did not show a significant worsening over time, suggesting that lipid peroxidation reaches an early steady state in this model. Instead, d-ROMs were higher in HFD versus NPD rats, with a progressive increase from T1 to T2 that was followed by a plateau at T3. This points to a dynamic and time-dependent elevation of circulating reactive oxygen metabolites and systemic hydroperoxide load, which appears to stabilize at later stages of disease progression. Our findings thus reveal a dissociation between early-established lipid peroxidation and a more gradually evolving oxidative burden, supporting the idea that redox imbalance represents a key downstream consequence of chronic metabolic dysfunction in HFD-induced obesity.

Considering the observed progressive metabolic and oxidative alterations induced by prolonged HFD exposure, our study aimed at elucidating the relationship between systemic dysmetabolism and brain-related functional alterations (Guadilla et al., 2021; Jang et al., 2024). To address this, our multidimensional approach was designed to disentangle partially dissociable relationships between metabolic alterations and behavioural outcomes across cognitive and affective domains.

Firstly, impairments were found in burrowing behaviour of HFD rats, an ethologically relevant measure of well-being and integrative brain function (Lippi, 2021; Pyndt Jørgensen et al., 2014; Deacon, 2006; Di Majo et al., 2024a; Koska et al., 2019). Although increased body mass may partially contribute to reduced burrowing efficiency, this behavioural alteration has been reported in HFD models (Di Majo et al., 2024a; Gambino et al., 2024; Pyndt Jørgensen et al., 2014), and is widely considered a sensitive indicator of compromised adaptive behaviour (Deacon, 2006), which progressively worsened over time in our study. Also, general locomotor activity and exploration appeared progressively reduced in HFD rats in the OFT. This decrease is likely attributable to obesity-related hypoactivity since increased body mass limits spontaneous locomotion, thus reflecting both motor and motivational constraints associated with weight gain rather than purely domain-specific deficits (Butler et al., 2025; Di Majo et al., 2024a; Gambino et al., 2024).

Alterations in affective dimension became particularly evident when assessed using paradigms specifically designed to probe anxiety-like behaviour. In the dark–light box, HFD rats exhibited gradually reduced time spent and entries in the illuminated compartment versus NPD rats, indicating a clear increase in anxiety-like behaviour as diet exposure progressed. While HFD rats did not show any alterations in feeding latency in the NSFT at the earliest timepoint, a marked and progressive increase in this parameter from T1 to T2 emerged with prolonged diet exposure, indicating a time-dependent modulation of the anxiety-like responses. In light of the progressive reduction in feeding behaviour observed in HFD animals, this factor should be taken into account when interpreting NSFT results, as altered motivational states may reflect an interaction between affective and motivational components rather than a purely anxiety-specific phenotype.

Overall, the robust anxiogenic phenotype emerging in the DWB and NSFT paradigms may indicate a progressive dysfunction of cortico–limbic circuits governing emotional salience and anxiety regulation, rather than a primary alteration of metabolic feeding drive, as also supported by a consistent trend toward reduced time spent in the centre in the OFT across time. In particular, these changes are consistent with altered amygdala responsivity, a reduced top–down inhibitory control exerted by the medial prefrontal cortex, and diet-induced neuroplastic and inflammatory alterations in the ventral hippocampus and in the hypothalamus, as evidenced also after chronic high-fat diet exposure (Boitard et al., 2014; Kanoski and Davidson, 2011; Mazzone et al., 2020; Sharma and Fulton, 2013; Vega-Torres et al., 2018). A critical aspect to consider is that all the behavioural paradigms discussed so far, including OFT, present a substantial emotional or anxiety-related component. In this light, we can speculate that prolonged HFD exposure disrupts emotional regulatory networks, leading to a progressive dominance of affective interference over goal-directed behaviour in aversive contexts.

Furthermore, specific and robust impairments emerged in the memory domain assessed via Object Recognition Test, revealing a marked reduction in the recognition of novel stimuli of HFD rats at both 1 h and 24 h retention intervals, with deficits becoming more pronounced at T2 and T3 versus T1. This indicates an impairment in declarative memory probably due to cognitive vulnerability that is in line with previous evidence from obesogenic diets disrupting hippocampal-dependent memory processes via dysregulation of neuromodulatory and neuroimmune signalling (Hayes et al., 2024; Liang et al., 2023). Findings from ORT, which is minimally influenced by anxiety-related components, support the interpretation of a genuine cognitive impairment, though future studies incorporating tasks with distinct emotional valence and reduced affective load will be essential to further disentangle specific domain-based contributions.

As far as the behavioural analyses outlined above reveal a clear temporal and dimensional progression of HFD-induced brain alterations across locomotor, cognitive and affective dimensions, the extent to which these changes are interrelated or how they evolve in concert with systemic dysmetabolism is not fully captured. Indeed, the coexistence of early cognitive impairments, progressively emerging affective disturbances and metabolic alterations raise a critical question as to whether these features can reflect coordinated trajectories within an expanding pathological neuro-metabolic phenotype.

To this purpose, an integrative longitudinal analytical framework was implemented in this study to jointly examine behavioural and metabolic domains and to identify which variables most closely tracked HFD phenotype progression. Through quantification of temporal dynamics and cross-domain associations within a multivariate space, this approach enabled the assessment of convergent and independent trajectories across metabolic and cognitive dimensions. Importantly, the causal statistical framework adopted here moves from descriptive associations to a putative mechanistic representation of how metabolic load propagates through neurometabolic mediators to shape cognitive systems.

Radar-based phenotypic mapping provided a direct visualization of disease progression. Control animals maintained a stable and compact phenotypic profile across time, whereas HFD rats exhibited a systematic outward expansion of the polygonal area from T1 to T3. Early expansion at T1 was driven primarily by metabolic variables (i.e. body weight, leptin, and ketone bodies) while cognitive and behavioural indices showed only modest displacement. By contrast, at later time points the expansion encompassed all dimensions, with pronounced shifts in anxiety-related behaviour and memory indices, indicating a temporal convergence of metabolic deterioration and cognitive dysfunction.

Principal Component Analysis further quantified this convergence. The first rotated component, accounting for nearly half of the total variance, was dominated by strong positive loadings of body weight, leptin, dROMS and glucose intolerance alongside negative loadings of memory performance and anxiety-related behaviour. This structure indicates that worsening cognitive performance scales along the same latent axis as increasing metabolic and redox burden. A second component tracked variance associated with lipid dysmetabolism and oxidation, with high loadings for triglycerides and ketone bodies, and inverse loadings for water and food intake, suggesting a partially independent metabolic dimension linked to energy expenditure and motivation.

On this basis we exploited causal modelling to unveil the directional structure within these associations. Our mediation analysis showed that body weight seems not to be directly involved in anxiety-related parameters assessed in DWB and NSFT paradigms. Instead, its influence on both outcomes was fully conveyed through indirect pathways mediated by systemic leptin signalling, as indicated by significant indirect effects in the absence of direct paths. Dysmetabolism alone seems indeed insufficient to explain behavioural alterations, whereas our mediated model supports the view that metabolic burden impacts cognitive and affective domains via intermediary neurometabolic processes. Above all, leptin neurotrophic and neuroplastic effects in physiological conditions were found to contribute to synaptic modulation and cognitive resilience (Zou et al., 2019). In accordance with this, chronic metabolic overload has been associated with central leptin resistance, in terms of altered receptor signalling and downstream neuromodulatory efficacy (O'Brien et al., 2021). This interpretation aligns with conceptual causal frameworks proposing leptin, rather than as a direct causal driver, as an indirect mediator between metabolic status and affective dimensions in human studies (Zhu et al., 2022). Nevertheless, leptin is unlikely to represent the sole biological mediator linking metabolic dysfunction to behavioural impairment, we acknowledge this limitation and highlight the assessment of other adipokines or metabolic mediators, such as adiponectin, insulin and FGF2, as important components for future studies aimed at further elucidating the biological mechanism at the basis of MetS-associated cognitive and affective decline.

In a complementary analysis, oxidative stress measured via dROMS levels was tested as well as an alternative pathway linking MetS to memory performance. The indirect effect of body weight on RI% via dROMS suggests a trend toward memory impairment that is preferentially conveyed through redox imbalance. This pathway appeared partially dissociated from leptin-mediated effects on anxiety-like behaviour, suggesting that multiple neurometabolic routes may underlie affective versus cognitive vulnerability in MetS, with oxidative stress exerting a particularly deleterious impact on hippocampal-dependent memory processes. Taken together, our multivariate and causal modelling analyses, though incapable of generating new mechanistic insights especially considering the limited sample size, however highlight the need for distinct domain-specific behavioural and biological markers when assessing brain dysfunction in MetS.

Nonetheless, we acknowledge that the present findings capture only a fraction of the complex molecular mechanisms underlying brain alterations associated with metabolic syndrome. Accordingly, future studies will be directed toward a more comprehensive investigation of dysregulated neuroinflammatory and neurotransmission pathways, with the aim of further clarifying the biological mechanisms linking metabolic dysfunction to cognitive impairment in MetS. In this view, progressive redox burden may represent a cumulative stressor on synaptic and network integrity, hence reducing cognitive performance as metabolic dysfunction advances (Naomi et al., 2023). Together, these data support the rationale that MetS-associated cognitive and affective impairments constitute an integral component of a progressively expanding neuro-metabolic phenotype, emerging early during disease evolution and dynamically interacting with systemic dysmetabolism.

## 5 Conclusions

In conclusion, cognitive and affective impairments appear to be embedded within the evolving architecture of the neuro-metabolic phenotype, emerging early and scaling with disease progression along defined mechanistic trajectories. While affective disturbances are more closely associated with leptin-related signalling pathways, cognitive deficits, particularly in declarative memory, appear to be preferentially linked to oxidative stress mechanisms, supporting a domain-specific organization of brain dysfunction in MetS. By integrating longitudinal behavioural and metabolic data within a unified computational framework, the present study provides evidence that cognitive vulnerability is integrated in, and contributes, to the temporal structure of MetS progression.

In this perspective, cognitive dysfunction may represent a quantifiable vulnerability dimension that precedes and potentially predicts adverse clinical trajectories associated with metabolic disorders. This framework therefore provides a basis for developing computational biomarkers of relapse risk and forecasting disease progression. Future translational studies should determine whether early modulation of neurometabolic signalling can influence the trajectory of cognitive and affective impairments in the clinical management of MetS.

## Funding

This work was supported with funds “Eurostart 2024” provided by Italian Ministry of University and Research (MUR) “FONDI PNR D.M. 737/2021" to Prof.Giuditta Gambino, CUP: B79J21038330001”.

## CRediT authorship contribution statement

**Nicolò Ricciardi:** Formal analysis, Investigation, Methodology, Writing – original draft. **Valentina Di Liberto:** Investigation, Methodology, Validation, Writing – original draft. **Danila Di Majo:** Investigation, Methodology, Writing – review & editing. **Antonio Cangelosi:** Formal analysis, Investigation, Software, Writing – review & editing. **Miriana Scordino:** Investigation, Writing – review & editing. **Giulia Urone:** Investigation, Writing – review & editing. **Giuseppe Giglia:** Supervision, Validation, Writing – review & editing. **Alessandro Massaro:** Investigation, Writing – review & editing. **Mario Allegra:** Methodology, Supervision, Validation, Writing – review & editing. **Maria Ankarcrona:** Resources, Supervision, Validation, Writing – review & editing. **Pierangelo Sardo:** Supervision, Validation, Writing – review & editing. **Giuseppe Ferraro:** Supervision, Validation, Writing – review & editing. **Giuditta Gambino:** Conceptualization, Data curation, Formal analysis, Funding acquisition, Investigation, Resources, Writing – original draft, Writing – review & editing.

## Declaration of competing interest

The authors declare that there are no conflicts of interest regarding the publication of this article. None of the authors has any financial or personal relationships that could inappropriately influence or bias the work reported in this manuscript.

All authors confirm that they have read and complied with the journal's policies on ethical publication and that this manuscript is consistent with those guidelines.

## Data Availability

Data will be made available on request.
