# Supplementary material for: Cognition at the core of metabolic syndrome: linking metabolic load to behavioural impairment in a longitudinal high-fat diet rat model
Source: Brain Behav Immun Health. 2026 Jun 16;55:101287. doi: 10.1016/j.bbih.2026.101287 (PMC13293659; doi:10.1016/j.bbih.2026.101287)
Supplement: Multimedia component 1 [file mmc1.docx]

Ricciardi N. - Cognition at the core

**Supplementary Materials**


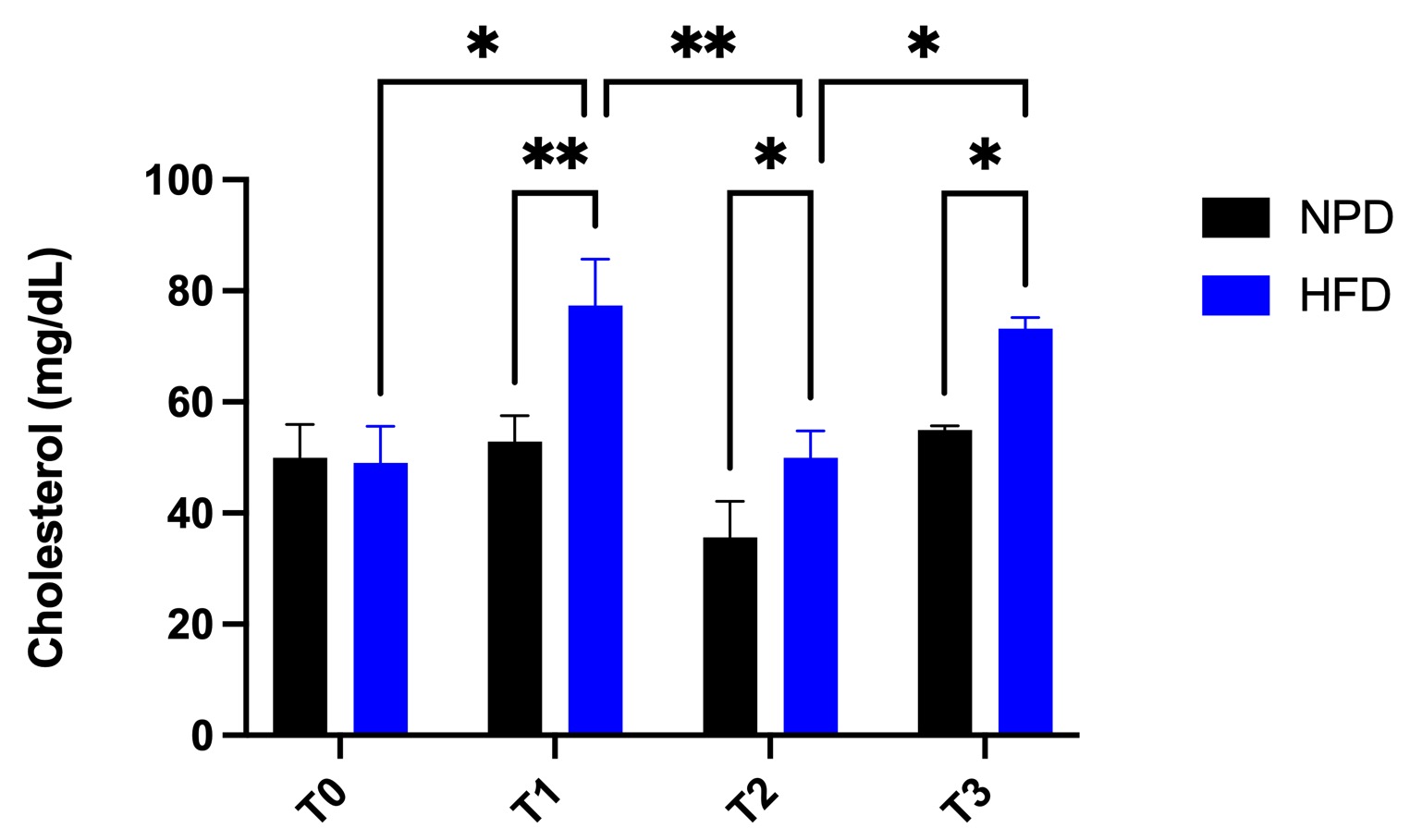


**Supplementary Figure 1.** Total cholesterol levels (mg/dL) in NPD and HFD groups at T0, T1, T2 and T3.

**
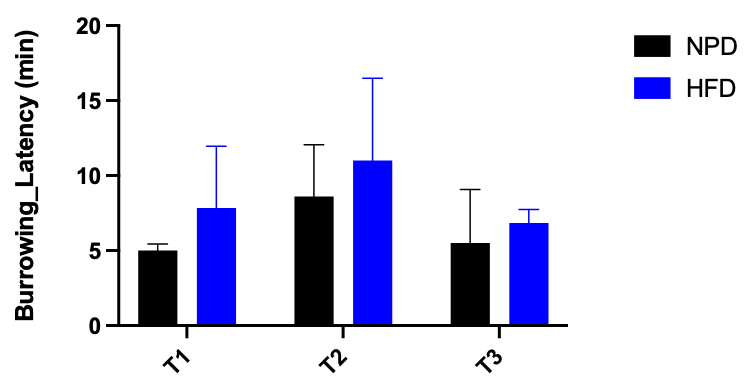
**

**Supplementary Figure 2.** Latency from first burrowing (min) assessed in the burrowing behaviour in NPD and HFD groups at T1, T2 and T3.


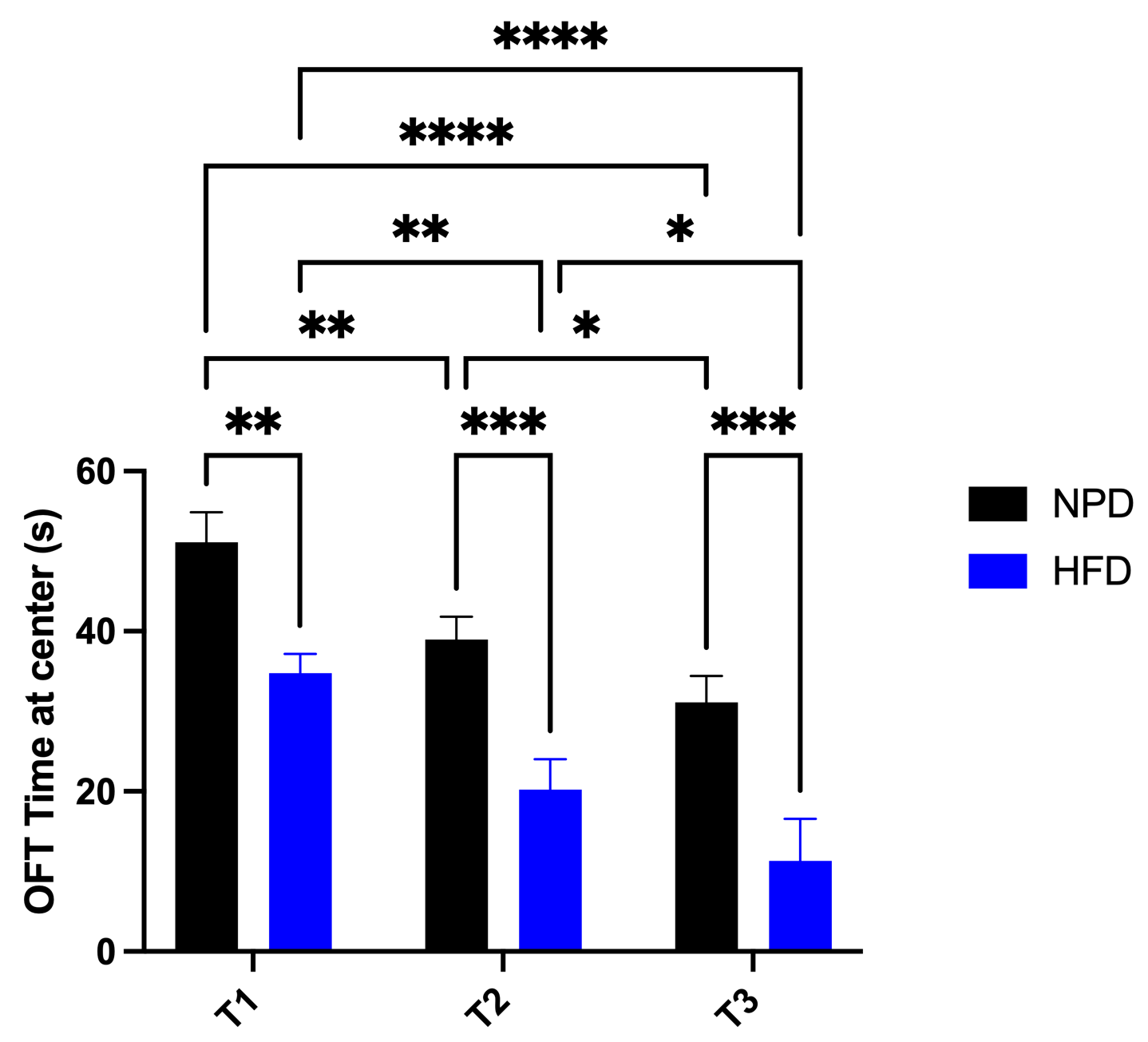


**Supplementary Figure 3.** Time spent in the centre zone (s) assessed in the Open Field Test in NPD and HFD groups at T1, T2 and T3. Statistical analysis was assessed by two-way ANOVA and significant Bonferroni post hoc tests are indicated by (*)p<0.05, (**)p<0.001, (***)p<0.0001 and (****)p*<*0.0001.


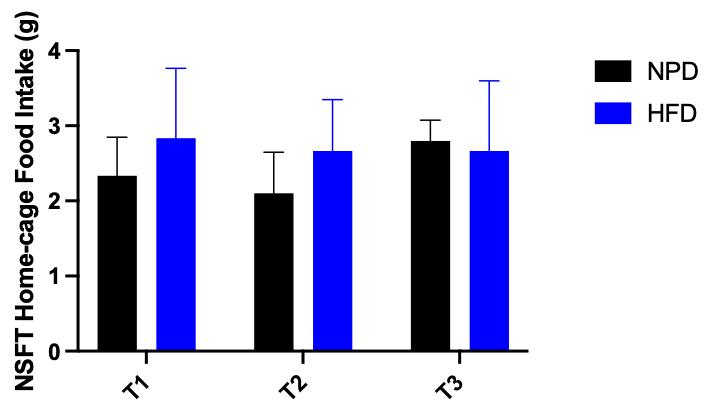


**Supplementary Figure 4.** Amount of food consumed once back in the domestic cage (Home-cage Food Intake) assessed in the Novelty-Suppressed Feeding test (NSFT) in the NPD and HFD groups at T1, T2 and T3.


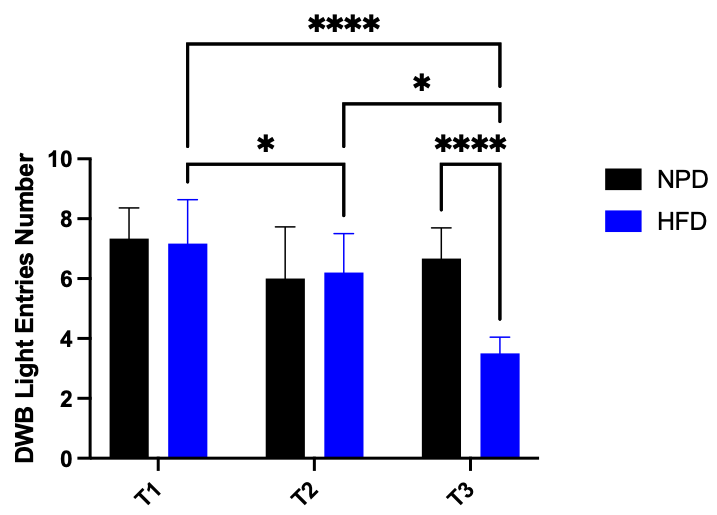


**Supplementary Figure 5.** Number of entrances to the light area assessed in the Dark-White Box (DWB) test in the NPD and HFD groups at T1, T2 and T3. Statistical significance by two-way ANOVA followed by Bonferroni post hoc tests is indicated by (*)p<0.05 and (****)p<0.0001.

**
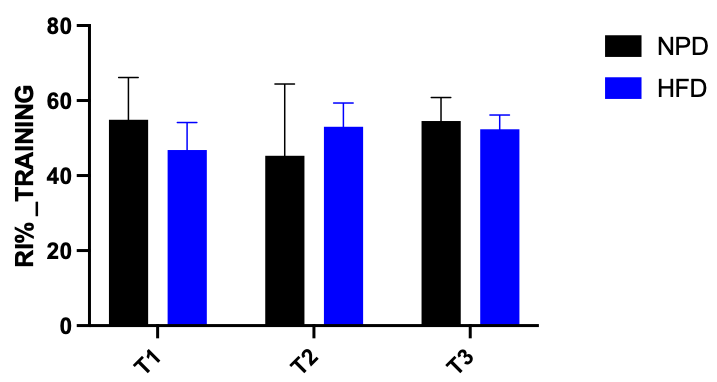
**

**Supplementary Figure 6.** (RI%) during the training phase of the Object Recognition Test evaluated in the NPD and HFD groups at T1, T2 and T3.

**A. Chi-Squared Test**

|  | **Value** | **df** | **p** |
| --- | --- | --- | --- |
| **Model** | 80.571 | 43 | < .001 |

**B. Component Loadings**

| **Variable** | **RC1** | **RC2** | **Uniqueness** |
| --- | --- | --- | --- |
| **Body Weight** | 0.950 |  | 0.183 |
| **Leptin** | 0.916 |  | 0.060 |
| **dROMS** | 0.867 |  | 0.266 |
| **AUC** | 0.798 |  | 0.345 |
| **DWB light time** | -0.731 |  | 0.476 |
| **RI% ret 1H** | -0.645 |  | 0.627 |
| **Food Intake** | -0.565 | -0.526 | 0.214 |
| **Water Intake** | 0.496 | -0.949 | 0.157 |
| **NSFT feeding time** | 0.450 |  | 0.596 |
| **TG** |  | 0.835 | 0.304 |
| **Ketones** |  | 0.707 | 0.363 |
| **LP-CHOLOX** |  | 0.565 | 0.621 |

**C. Component Characteristics**

| **Component** | **Eigenvalue (Unrotated)** | **Proportion var. (Unrotated)** | **Cumulative (Unrotated)** | **SumSq. Loadings (Rotated)** | **Proportion var. (Rotated)** | **Cumulative (Rotated)** |
| --- | --- | --- | --- | --- | --- | --- |
| **Component 1** | 5.419 | 0.452 | 0.452 | 4.887 | 0.407 | 0.407 |
| **Component 2** | 2.367 | 0.197 | 0.649 | 2.899 | 0.242 | 0.649 |

**Supplementary Table 1.** Principal Component Analysis (PCA) of Metabolic and behavioural Variables. **A.** Model Fit and Chi-Squared Test. The table reports the chi-squared value, degrees of freedom, and p-value for the overall model validation. **B.** Component Loadings and Uniqueness. The table reports rotated component loadings (RC1 and RC2) and uniqueness values for all metabolic, dietary, and oxidative stress variables following Promax rotation. **C.** Component Characteristics. The table reports eigenvalues, proportion of variance, and cumulative variance for both unrotated and rotated solutions (see Figure 8).

**A. Path coefficients**

| **Path** | **Estimate** | **Std. Error** | **z-value** | **p** | **95% CI Lower** | **95% CI Upper** |
| --- | --- | --- | --- | --- | --- | --- |
| **Body Weight → DWB light time** | -0.201 | 0.232 | -0.868 | 0.386 | -0.655 | 0.253 |
| **Leptin → DWB light time** | -0.573 | 0.263 | -2.174 | 0.030 | -1.089 | -0.057 |
| **Body Weight → Leptin** | 0.680 | 0.132 | 5.167 | < .001 | 0.422 | 0.938 |
| **Body Weight → NSFT feeding time** | -0.102 | 0.310 | -0.327 | 0.743 | -0.710 | 0.507 |
| **Leptin → NSFT feeding time** | 0.808 | 0.353 | 2.290 | 0.022 | 0.117 | 1.500 |
| **Body Weight → RI% ret 1H** | 0.028 | 0.329 | 0.085 | 0.933 | -0.618 | 0.673 |
| **Leptin → RI% ret 1H** | -0.442 | 0.374 | -1.180 | 0.238 | -1.176 | 0.292 |

**B. Direct and indirect effects**

| **Path** | **Estimate** | **Std. Error** | **z-value** | **p** | **95% CI Lower** | **95% CI Upper** |
| --- | --- | --- | --- | --- | --- | --- |
| **Body Weight → DWB light time** | -0.201 | 0.232 | -0.868 | 0.386 | -0.655 | 0.253 |
| **Body Weight → NSFT feeding time** | -0.102 | 0.310 | -0.327 | 0.743 | -0.710 | 0.507 |
| **Body Weight → RI% ret 1H** | 0.028 | 0.329 | 0.085 | 0.933 | -0.618 | 0.673 |
| **Body Weight → Leptin → DWB light time** | -0.390 | 0.194 | -2.004 | 0.045 | -0.770 | -0.009 |
| **Body Weight → Leptin → NSFT feeding time** | 0.549 | 0.262 | 2.094 | 0.036 | 0.035 | 1.064 |
| **Body Weight → Leptin → RI% ret 1H** | -0.300 | 0.261 | -1.150 | 0.250 | -0.812 | 0.211 |

**C. Total effects**

| **Type** | **Path** | **Estimate** | **Std. Error** | **z-value** | **p** | **95% CI Lower** | **95% CI Upper** |
| --- | --- | --- | --- | --- | --- | --- | --- |
| Total | Body Weight → DWB light time | -0.591 | 0.165 | -3.574 | < .001 | -0.915 | -0.267 |
| Total | Body Weight → NSFT feeding time | 0.448 | 0.224 | 2.001 | 0.045 | 0.009 | 0.886 |
| Total | Body Weight → RI% ret 1H | -0.273 | 0.217 | -1.256 | 0.209 | -0.698 | 0.153 |
| Total indirect | Body Weight → DWB light time | -0.390 | 0.194 | -2.004 | 0.045 | -0.770 | -0.009 |
| Total indirect | Body Weight → NSFT feeding time | 0.549 | 0.262 | 2.094 | 0.036 | 0.035 | 1.064 |
| Total indirect | Body Weight → RI% ret 1H | -0.300 | 0.261 | -1.150 | 0.250 | -0.812 | 0.211 |

**Supplementary Table 2.** Path Analysis and Mediation Models for Body Weight, Leptin, and behavioural Outcomes. **A.** Path coefficients. The table reports parameter estimates, standard errors, z-values, p-values, and 95% confidence intervals for the direct paths between body weight, leptin, and behavioural variables including DWB light time, NSFT feeding time, and RI% ret 1H. **B.** Direct and indirect effects. The table reports parameter estimates, standard errors, z-values, p-values, and 95% confidence intervals for the direct influence of body weight on behavioural outcomes and the indirect pathways mediated through leptin levels. **C.** Total effects. The table reports parameter estimates, standard errors, z-values, p-values, and 95% confidence intervals for the total combined effects and total indirect effects of body weight across all specified behavioural models. (see **Fig. 6**).

**A. Path Coefficients**

| **Predictor** |  | **Outcome** | **Estimate** | **Std. Error** | **z-value** | **p** | **Lower (95% CI)** | **Upper (95% CI)** |
| --- | --- | --- | --- | --- | --- | --- | --- | --- |
| Body Weight | → | RI% ret 1H | -0.200 | 0.200 | -0.999 | .318 | -0.593 | 0.193 |
| dROMS | → | RI% ret 1H | -0.360 | 0.194 | -1.850 | .064 | -0.741 | 0.021 |
| Body Weight | → | dROMS | 0.690 | 0.130 | 5.321 | < .001 | 0.436 | 0.943 |

**B. Direct and Indirect Effects**

| **Type** | **Predictor** |  | **Outcome** | **Estimate** | **Std. Error** | **z-value** | **p** | **Lower (95% CI)** | **Upper (95% CI)** |
| --- | --- | --- | --- | --- | --- | --- | --- | --- | --- |
| Total | Body Weight | → | RI% ret 1H | -0.448 | 0.156 | -2.871 | .004 | -0.754 | -0.142 |
| Total indirect | Body Weight | → | RI% ret 1H | -0.248 | 0.142 | -1.748 | .081 | -0.526 | 0.030 |

**C. Total Effects**

| **Type** | **Predictor** |  | **Outcome** | **Estimate** | **Std. Error** | **z-value** | **p** | **Lower (95% CI)** | **Upper (95% CI)** |
| --- | --- | --- | --- | --- | --- | --- | --- | --- | --- |
| Total | Body Weight | → | RI% ret 1H | -0.448 | 0.156 | -2.871 | .004 | -0.754 | -0.142 |
| Total indirect | Body Weight | → | RI% ret 1H | -0.248 | 0.142 | -1.748 | .081 | -0.526 | 0.030 |

**Supplementary Table 3.** Path Analysis and Mediation Model for Body Weight, dROMS, and RI% ret 1H. **A.** Path coefficients. The table reports parameter estimates, standard errors, z-values, p-values, and 95% confidence intervals for the direct associations between body weight, oxidative stress markers (dROMS), and behavioural outcomes (RI% ret 1H). **B.** Direct and indirect effects. The table reports parameter estimates, standard errors, z-values, p-values, and 95% confidence intervals for the direct influence of body weight on RI% ret 1H and the mediated pathway through dROMS. **C.** Total effects. The table reports parameter estimates, standard errors, z-values, p-values, and 95% confidence intervals for the combined total effect and the total indirect effect of body weight on the target behavioural variable.
